# Death from a Dose of Urine

**Published:** 1852-08

**Authors:** 


From the Buffalo Medical Journal.

*Death from a Dose of Urine.*—Dr. Flint.

You know that various animal excrements have always had a nursery reputation. Thus, human saliva, urine, and fæces, contend with hen's dung, sheep's dung, and cow's dung, in the esteem of many excellent mothers and nurses. More of these disgusting mixtures are pushed down the throats of unoffending infants every day than it were decent to expose. You will learn by the following case that human urine cannot always be given with impunity:

An infant, aged eight weeks, was strong and healthy. In the night she became restless and cried, and the mother, to make her quiet, gave her a table-spoonful and a half of undiluted urine. When she took the child up it laughed. Ten minutes after it was comatose; and in fifteen minutes became convulsed. It continued to convulse and to froth at the mouth about eight hours, when it died.

The family were very poor, and no physician was called.

Yours truly, X. Y. Z.

